# Disentangling the influence of climate, soil and belowground microbes on local species richness in a dryland ecosystem of Northwest China

**DOI:** 10.1038/s41598-017-17860-7

**Published:** 2017-12-21

**Authors:** Jianming Wang, Ting Long, Yueming Zhong, Jingwen Li, Tianhan Zhang, Yiming Feng, Qi Lu

**Affiliations:** 1 College of Forestry, Beijing Forestry University, No. 35 Qinghua East Road, Haidian District, Beijing, 100083 China; 2 Institute of Desertification Studies, Chinese Academy of Forestry, NO.10 Huaishuju Road, Haidian District, Beijing, 100091 China

## Abstract

Xinjiang Uygur Autonomous Region (XUAR) covers one of the largest drylands in the world, while the relative effects of different environmental factors on plant diversity are poorly understood. We sampled 66 sites in a typical dryland of XUAR, which covers more than 450,000 km^2^, to evaluate the relative influences of different factors on the patterns of local plant species richness (LPSR). We found that overall and herbaceous LPSR were positively correlated with water availability, soil nutrients but negatively correlated with energy availability, while the shrub LPSR showed the opposite response. Climate, soil attributes together explained 53.2% and 59.2% of the variance in overall and herbaceous LPSR, respectively; revealing that LPSR patterns were shaped by abiotic and underground biotic factors together. Only 31.5% of the variance in the shrub LPSR was explained by soil attributes, indicating that shrub LPSR was mainly limited by non-climatic factors. There findings provide robust evidence that relative contribution of climate and soil attributes differ markedly depending on the plant functional group. Furthermore, we found the different relationship between microbes and plant diversity, indicating that the linkages between soil microbial diversity and plant diversity may vary across functional groups of microbes and plant. These findings provide robust evidence that the relative roles of climate, soil and microbes differ markedly depending on the plant functional group. Microbial richness showed a significantly pure influence on the LPSR of all groups, suggesting that microbes play a non-negligible role in regulating plant diversity in dryland ecosystems.

## Introduction

Diversity patterns are distributed heterogeneously over broad spatial scales^1^. As one of the most remarkable patterns in biology, changes in plant species diversity across environmental gradients have intrigued biologists for at least two centuries^2–4^. In recent decades, ecologists have proposed many hypotheses to identify the environmental factors controlling geographic patterns in plant diversity. Among them, the contemporary climate hypothesis has been supported by the majority of studies. Current climatic conditions often account for more than 80% of the variation in species richness, and climate has therefore been regarded as the primary predictor of plant diversity by some authors^5–7^. However, climate-richness relationships may vary geographically, and no consensus has been reached at the global scale^8,9^. For instance, water availability accounts for more variation in species richness in areas with high energy inputs^10^, while energy is more important in cold regions^11^.

Although climate is considered one of the most important environmental factors determining spatiotemporal patterns of plant diversity and community composition at multiple scales^11,12^, other local environmental factors, such as soil attributes and biotic factors, also exert significant influence^8,13–18^. Numerous studies conducted in diverse regions and ecosystems have found that soil attributes significantly influence species richness^17–22^. For example, compared to habitats with more homogeneous soils, habitats with heterogeneous soils are thought to support more species^21^. In South America, soil fertility and climate have been considered the most significant abiotic predictors of plant diversity^15,20^. Further, several studies have indicated that species diversity increases with soil fertility in semi-arid environments^21,22^, and soil heterogeneity has been reported to have an important effect on species richness in dry calcareous grassland^23^. However, similar to climate-richness relationships, the relationship between soil and species richness also differs geographically, with no unified understanding.

All terrestrial ecosystems are composed of belowground and aboveground portions, and their interactions affect ecological processes and properties at the community and ecosystem levels^24,25^. Exploring the associations between the belowground and aboveground biotic communities may shed new understanding of the maintenance of biodiversity. Soil microbes play a crucial role in the vast majority of fundamental ecosystem processes^25,26^, including nutrient acquisition^27^, nitrogen and phosphorus^28,29^ cycling and soil formation^30^. Moreover, there is increasing evidence that belowground microbial diversity significantly correlated with plant diversity. However, the relationship between microbial diversity and plant diversity vary among different regions and ecosystems and, importantly, may be positively, negatively or no related. Hence, the different associations between the belowground and aboveground biotic communities need be examined. Arbuscular mycorrhizal (AM) fungi can directly enhance plant diversity: they have been shown to increase species richness by nearly 30% in European grassland^25,31^, and several studies also found that AM fungi can decrease species diversity in tallgrass prairie^32^. It has been emphasized that soil symbiotic bacteria and pathogens can significantly affect the composition and diversity of the plant community^33,34^. However, recent evidence also showed no obvious association between soil bacteria and plant species diversity in early successional temperate forests^35^.

The determinants of plant diversity may differ significantly among different plant functional types^11,36^. Comparisons among ecological or taxonomic plant types are necessary to gain a more comprehensive understanding of diversity patterns^37,38^. However, our understanding of the differences in the determinants of diversity for different groups remain limited.

The Xinjiang Uygur Autonomous Region (XUAR) covers one of the largest drylands in the world. An abundant flora, nutrient-rich soil and climatic gradients occur in the typical dryland ecosystem in northern XUAR because of its unique mountain-basin system. To date, studies on plant diversity along environmental gradients in the dryland of XUAR have mainly focused on climate^10^, while the local factors such as soil attributes, especially belowground microbes remain poorly understood. Furthermore, the relative contributions of these factors to local plant species richness (LPSR) have seldom been quantified. In this paper, we sampled 66 sites in a typical dryland ecosystem in XUAR to address three specific questions: (1) What are the linkages between plant diversity and microbial diversity in a typical dryland ecosystem in Northwest China? (2) What are the relative influences of climate and soil attributes on the geographic patterns of LPSR? (3) Are the patterns similar for different plant functional types?

## Materials and Methods

### Study area

**Figure 1:**
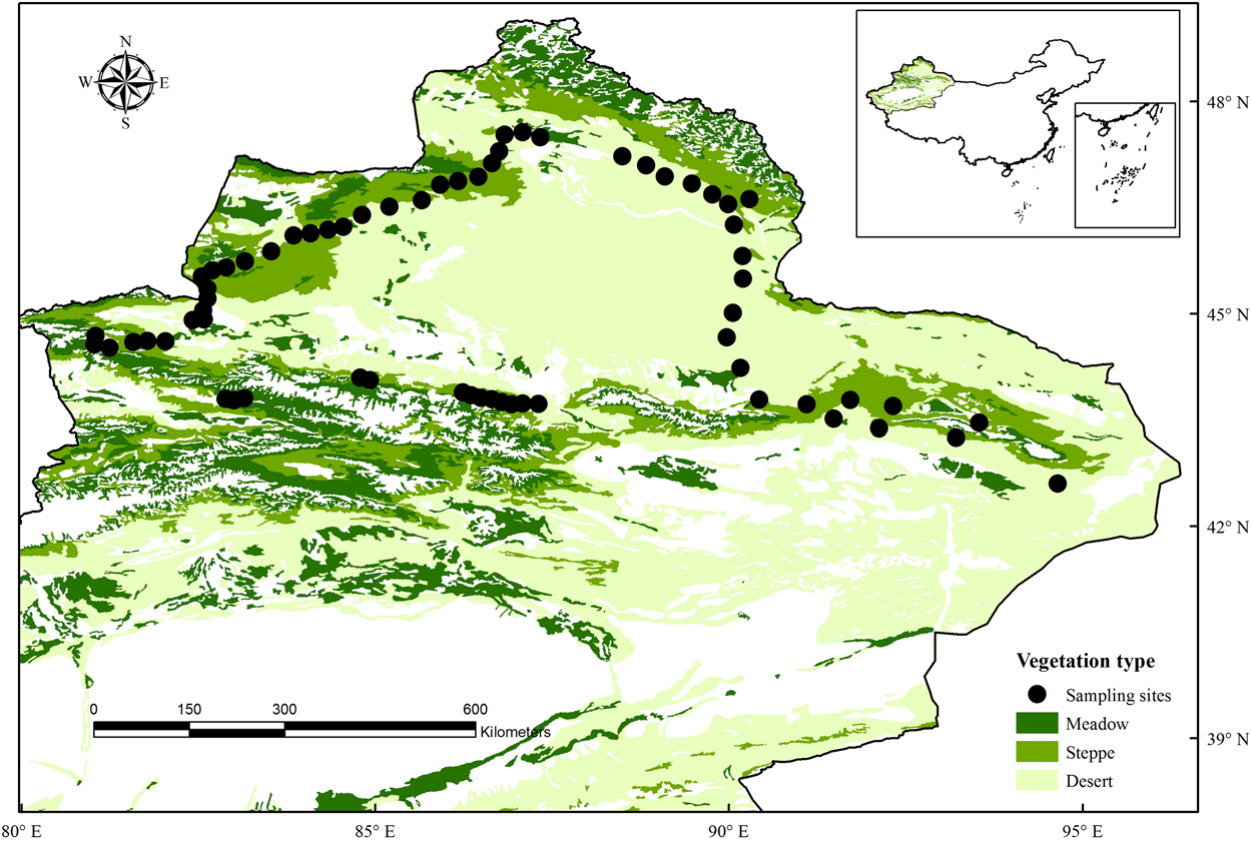
Locations of the study sites in typical dryland of Northwest China. Our sampling scheme was designed to explore the geographic patterns of plant species in a typical dryland ecosystem, which spans dry-subhumid, semi-arid and arid ecosystems, as demonstrated in the legend. The dataset was provided by the Data Center for Resources and Environmental Sciences, Chinese Academy of Sciences (RESDC) (http://www.resdc.cn), and the maps were created using ArcGIS 10 (http://www.esri.com/software/arcgis).

**Figure 2:**
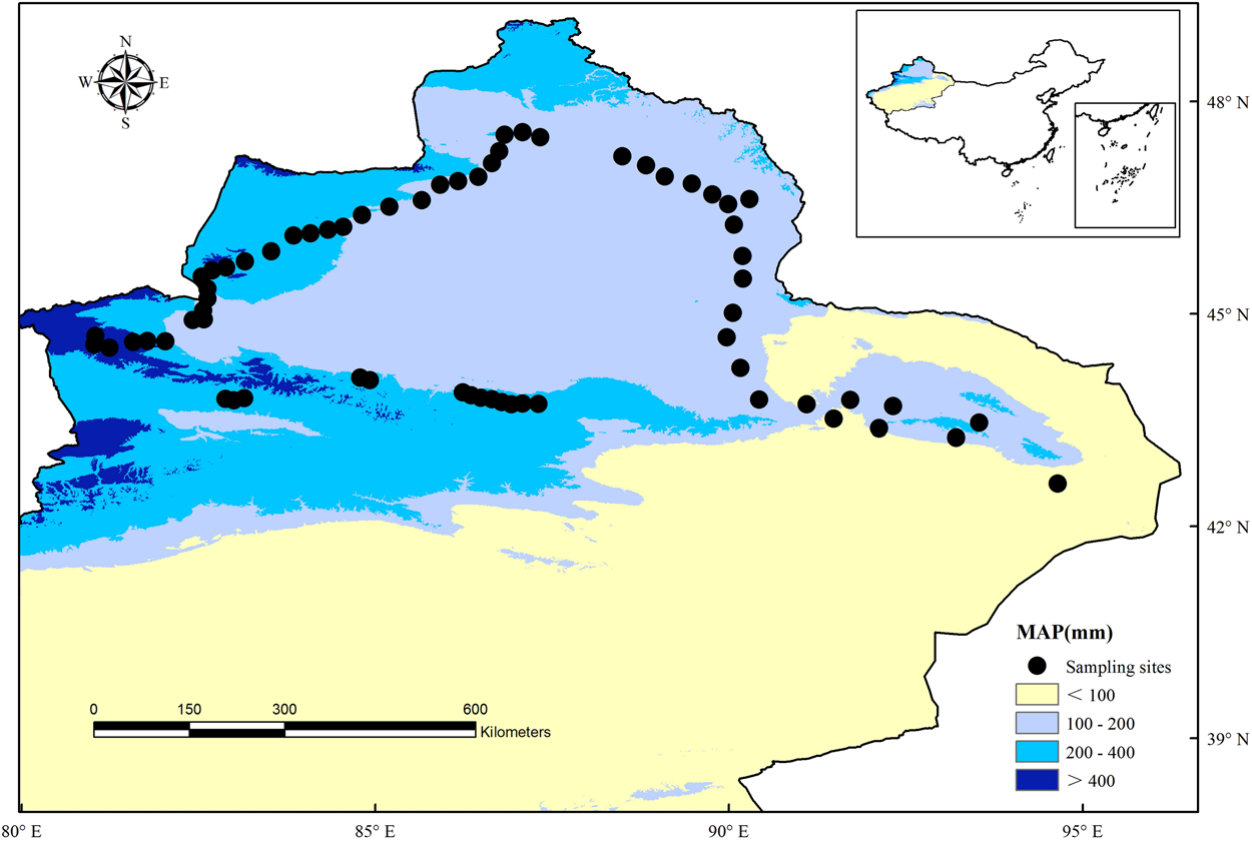
Mean annual precipitation (MAP) gradient map of the study area. Mean annual precipitation is shown as the background to the sites sampled in our study. The Mean annual precipitation dataset was provided by the WorldClim global climate database (http://www.worldclim.org), and the maps were created using ArcGIS 10 (http://www.esri.com/software/arcgis).

We sampled 66 sites in a typical dryland ecosystem (the region includes dry-subhumid, semi-arid and arid ecosystems^39^) in XUAR during the peak of the growing season (July–August) of 2016 (Fig. 1). XUAR, one of the largest drylands in the world, is located in Northwest China and has a total area of approximately 1,640,000 km^2^. The general topographic features can be characterized as basins (e.g., the Junggar Basin and Tarim Basin; Fig. 2) separated by three longitudinal mountain systems (i.e., the Tianshan Mountains, Altay Mountains, and Kunlun Mountains; Fig. 2), and the altitude ranges from 156 m to 8611 m from the low basins to high mountains^11^. The climate is mainly controlled by a polar continental air mass, ranging from extreme arid and arid to semi-arid and semi-humid zones from east to west and from plateau subfrigid and plateau temperate to warm temperate and temperate zones from south to north. The climate is mainly arid to semi-arid, with high variability of precipitation and temperature. Due to its unique mountain-basin system and large climatic gradients, XUAR has various vegetation types, shifting from desert-steppe to forest-meadow from the low basins to high mountains^10^.

### Vegetation investigation and soil sampling

At each site, a 100 m^2^ plot was established in an area with typical dryland vegetation, within which all vascular plants were recorded and classified as shrubs or herbaceous plants. We defined the total number of plant species recorded in the plot as the LPSR. Meanwhile, geographical factors (latitude and longitude) and topographic factors (altitude, slope, aspect and slope position) were measured and recorded in each plot during the vegetation investigations. Within each plot, soil samples were collected from 15 randomly selected points (0–10 cm depth) where dominant perennial and non-perennial or other plant species were distributed and then mixed together into a single sample. The well-mixed soil samples were sieved using 2 mm mesh and subdivided into two portions; the first portion was stored in a thermally insulated box for the determination of soil physicochemical properties, and the other was stored at −20 °C prior to DNA extraction.

Soil total nitrogen (TSN) and soil available nitrogen (AN) was determined by the Kjeldahl procedure and Alkali diffusion method, respectively. Total soil organic matter (SOM) and total phosphorus (TSP) were determined using K2Cr2O7 oxidation and molybdenum blue method, respectively. Soil moisture content (SM) was measured gravimetrically after drying soil in an oven at 105 °C for 48 h. Finally, soil pH was determined in a 1:2.5 ratio of fresh soil to water slurry. Soil sand content(SC) and clay content (CC) were measured on a particle size analyzer after removal of organic matter and calcium carbonates using H_2_O_2._

### DNA extraction, PCR amplification and Illumina MiSeq sequencing

We extracted genomic DNA from the fresh soil samples (0.5 g) using E.Z.N.A. soil DNA kits (OMEGA, USA) following the manufacturer’s instructions. All extracted DNA samples were stored at −40 °C for subsequent analysis.

To assess the bacterial and fungal communities, we amplified the V3–V4 hypervariable region of the bacterial 16 S rRNA gene (using the forward primer 338 F (5′-ACTCCTACGGGAGGCAGCAG-3′) and the reverse primer 806 R (5′-GGACTACHVGGGTWTCTAAT-3′)) and the fungal ITS regions (using the forward primer ITS1-F (5′-CTTGGTCATTTAGAGGAAGTAA-3′) and the reverse primer ITS2 (5′-TGCGTTCTTCATCGATGC-3′)). These primers contained a set of 8-nucleotide barcode sequences unique to each sample. PCR amplifications were performed following the procedure described previously^40^.

PCR products were pooled and purified using the Agarose Gel DNA purification kit (Axygen Biosciences, Union City, CA, U.S.). The purified PCR products were pooled in equimolar concentrations and paired-end sequenced (2 × 300) on an Illumina MiSeq platform according to the standard protocols.

### Bioinformatics analysis

The high-quality sequence data were processed following the procedure described previously^40–42^ using the QIIME package (Quantitative Insights Into Microbial Ecology) (v1.2.1). The unique sequence set was classified into operational taxonomic units (OTUs) under the threshold of 97% identity using UCLUST. Chimeric sequences were identified and removed using Usearch (version 8.0.1623). The taxonomy of each 16S rRNA gene sequence was analysed against the Silva119 16S rRNA database using UCLUST with a confidence threshold of 90%, while the taxonomy of each ITS gene sequence was analysed by comparison against sequences within the Unite 7.0 database using UCLUST. The richnesses of total bacteria, total fungi and trophic groups (i.e., saprotrophs (SSR), pathogens (PR), animal parasites (APR), ectomycorrhizae (EMR) and arbuscular mycorrhizae (AMR)) were used as variables to examine the influence of underground microbes on LPSR in our study. Richness was calculated using subsets of the same sequences, which were randomly selected from each sample^43^. Furthermore, the richness of fungal trophic groups was calculated following criteria described previously^44,45^ (details in Table S2).

### Climate data

We selected six climate variables that are often considered to influence plant species diversity^5–9^, including (1) water factors: mean annual summer precipitation (MASP) and actual evapotranspiration (AET) and (2) energy factors: mean annual temperature (MAT), mean temperature of the coldest month (MTCM), mean temperature of the warmest month (MTWM), and potential evapotranspiration (PET). MASP, MAT, MTCM and MTWM were calculated using the monthly mean temperature and precipitation data for each site. The monthly mean temperature and precipitation data were extracted from the WorldClim global climate database using the geographic coordinates for each site (http://www.worldclim.org, with a resolution of 1 km × 1 km). We obtained PET and annual AET data from the website of the Consortium for Spatial Information (CGIAR-CSI) of the Consultative Group on International Agricultural Research (http://www.cgiar-csi.org, with a resolution of 1 km × 1 km).

### Statistical analyses

The analysis used the overall, shrub, and herbaceous LPSR values and three groups of environmental variables: 1) climate (MASP, MAT, MTCM, MTWM, AET, PET), 2) soil attributes (PH, TSN, TSPAN, SC, CC), and 3) microbial diversity (total bacteria, total fungi and trophic group richness) (Table 1). Prior to analysis, AET, TSN, SOM, SC, SSR, PR were log transformed, while AN and CC were square root transformed. To account for zero similarity values, AMR, EMR, and APR were log (x + 1) transformed. For Shrub plant, because 9 plots have no shrub no shrubs recorded, we only choose the climatic, soil and microbial variables and shrub LPSR of 57 plots (delete 9 plots that no shrubs recorded).

**Table 1:** Descriptive statistics of LPSR and environmental factors in our study. Some factors were square root (**) or log (*) transformed before analysis.

	Max	Mean	Min	SD	Skewness	Kurtosis
Local richness (/plot)						
Overall	21	11.39	5	3.26	0.45	0.39
Shrub	6	2.65	1	1.35	0.72	0.15
Herbaceous	15	7.50	1	3.22	−0.37	0.29
Geographic variables						
Altitude (m)	2153	1193.27	216	460.36	−0.01	−0.31
Longitude (°E)	94.65	86.55	81.04	3.55	0.3	−0.9
Latitude (°N)	47.56	45.2	42.6	1.36	0.14	−1.31
Climate						
MASP (mm)	237	108.52	32	45.36	1.05	0.91
MAT (°C)	8.64	4.2	−0.58	2.21	−0.17	−0.22
AET (mm)*	400	186.5	46	71.38	1.31	2.23
PET (mm)	1064	881.47	698	91.17	0.02	−0.03
MTCM (°C)	−10.6	−15.17	−19.4	2.29	−0.32	−1.05
MTWM (°C)	25.7	20.42	14.2	2.76	−0.34	−0.04
Soil						
SM (%)*	0.23	0.013	−8.22	0.038	3.99	17.38
SOM (g/kg)*	113.87	25.96	1.21	27.96	1.52	1.46
TSN (g/kg)*	6.14	1.48	0.11	1.46	1.36	0.93
TSP (g/kg)	1.03	0.59	0.31	0.16	0.58	0.3
Available N (mg/kg)**	116.78	30.36	1.39	26.44	1.55	1.81
pH	9.54	8.07	5.99	0.73	−0.63	0.85
SC (%)*	88	54.15	30	13.61	0.33	−0.22
CC (%)**	48	18.64	2	10.42	0.88	0.73
Belowground microbes						
Soil bacterial richness	1602	1209.9	412	243.2	−0.97	1.15
Soil fungal richness						
Total fungal richness	885	600.6	299	137.68	0.08	−0.54
SSR*	311	187.30	85	38.26	0.98	0.39
AMR*	77	17.50	0	16.38	2.26	1.48
RMR*	31	7.85	0	6.69	2.22	1.36
PR*	65	43.74	20	10.66	−0.65	0.07
APR*	11	3.61	0	1.92	2.84	1.24

Abbreviations: SD, standard deviation; SM, soil moisture content; pH, soil pH; TSN, total nitrogen; TSP, total phosphorus; SOM, soil organic matter; AN, available nitrogen; SC, sand content; CC, clay content; MASP, mean annual summer precipitation; AET, actual evapotranspiration; MAT, mean annual temperature; PET, potential evapotranspiration; MTWM and MTCM, mean temperature of the coldest month and the warmest month, respectively; SSR, saprotrophic richness; PR, pathogenic richness; APR, animal parasitic richness; EMR, ectomycorrhizal richness; AMR, arbuscular mycorrhizal richness.

First, the relationship between LPSR and geographic factors was explored using ordinary least square (OLS). Then we explored the relationships between LPSR and climate, soil attributes or microbial diversity by ordinary least square (OLS) regressions. Second, multiple regression analysis was used to evaluate the influence of different environmental variables on LPSR. To prevent data overfitting, different variables were subjected to forward-selection until *P*adj < 0.05 for all variables within the ‘vegan’ package^46^.

Finally, we conducted a partial regression analysis with a redundancy analysis (RDA) using the ‘vegan’ package^46^ to examine the relative impacts of climate, soil attributes and underground microbes on LPSR. In our study, the variation in LPSR was decomposed into the following four fractions^47^: (1) c and a was the pure influences of climate, soil attributes, respectively; (2) b as the joint influences of soil attributes and climat; and (3) unexplained variation. We also tested the significance of the pure effects of climatic and edaphic factors (c and a, respectively).

All quantitative analyses were conducted using R 3.3.2 (R Development Core Team).

## Results

### The geographical distribution patterns of LPSR

Our investigation identified a total of 244 species of vascular plants (Table S1) that could be classified into 39 families and 137 genera. Only 47 shrub species were recorded (belonging to 14 families and 28 genera), while 197 herbaceous species were found (35 families and 114 genera). The observed numbers of overall, shrub, and herbaceous species ranged from 5 to 21, 0 to 6 and 1 to 15, respectively (Table 1).

The correlation analysis showed that the overall and herbaceous LPSR increased with increasing altitude but decreased with increasing longitude (*P* <0.05; Table 2), while the shrub LPSR was uncorrelated with altitude and longitude (*P* > 0.05). Moreover, the overall and shrub and herbaceous LPSR showed no significant response to latitude (*P* > 0.05).

**Table 2:** The relationship between local species richness and geographic variables.

Richness	Altitude		Longitude		Latitude	
	r	*P*	r	*P*	r	*P*
Overall (n = 66)	0.52	<0.001	−0.32	0.008	−0.21	0.096
Shrub (n = 57)	−0.06	0.664	0.22	0.094	0.087	0.519
Herbaceous (n = 66)	0.55	<0.001	−0.36	0.003	−0.23	0.059

### Relationships between LPSR and climate, soil attributes and microbes

The overall and herbaceous LPSR linearly increased with the AET, MASP, SM, TSN, SOM, and CC (all *P* < 0.05; Table 3) and linearly decreased with MAT, PET, MTWM, MTCM, SC, and pH (all *P* < 0.05). However, the LPSR of shrub species was positively correlated with the MAT, PET, MTWM, MTCM, SC, and pH (all *P* < 0.05) and negatively correlated with the AET, MASP, SM, TSN, SOM, and CC (all *P* < 0.05). Furthermore, the overall, shrub and herbaceous LPSR all showed no significant response to TSP (all *P* > 0.05).

**Table 3:** Relationships between different environmental factors and LPSR.

	All species (n = 66)	Shrub species (n = 57)	Herbaceous species (n = 66)
	R^2^	R^2^	R^2^
Climate			
MASP	0.447(+)***		0.465(+)***
MAT	0.239(−)***		0.261(−)***
Log(AET)	0.323(+)***	0.096(−)*	0.359(+)***
PET	0.387(−)***		0.358(−)***
MTCM	0.082(−)*		0.108(−)**
MTWM	0.341(−)***		0.376(−)***
Soil			
Log(SM)	0.265(+)***		0.295(+)***
Log(TSN)	0.367(+)***	0.160(−)**	0.487(+)***
TSP			
Log(SOM)	0.425(+)***	0.085(−)**	0.488(+)***
Sqr(AN)	0.153(+)**		0.230(+)***
pH	0.163(−)***		0.207(−)***
Log(SC)	0.202(−)***	0.126(+)**	0.288(−)***
Sqr(CC)	0.246(+)***	0.161(−)**	0.413(+)***
Belowground microbes			
Soil bacterial richness	0.276(+)***		0.226(+)***
Soil fungal richness			
Total fungi	0.167(+)***	0.130(−)**	0.106(+)**
Log(SSR)	0.084(+)*		
Log(AMR + 1)	0.193(+)**	0.225(−)**	0.189(+)***
Log(EMR + 1)	0.079(−)*	0.101(+)*	0.062(−)*
Log(PR)	0.117(+)**		
Log(APR + 1)			

We defined the relationships between different environmental factors and species richness as follows: positive (+), negative (−). *P*-values are reported if their significance level is below 0.05. *P ≤ 0.05, **P ≤ 0.01 and ***P ≤ 0.001. SM, soil moisture content; TSN, total nitrogen; TSP, total phosphorus; SOM, soil organic matter; AN, available nitrogen; SC, sand content; CC, clay content; MASP, mean annual summer precipitation; AET, actual evapotranspiration; MAT, mean annual temperature; PET, potential evapotranspiration; MTWM and MTCM, mean temperature of the coldest month and the warmest month, respectively; SSR, saprotrophic richness; PR, pathogenic richness; APR, animal parasitic richness; EMR, ectomycorrhizal richness; AMR, arbuscular mycorrhizal richness.

**Figure 3:**
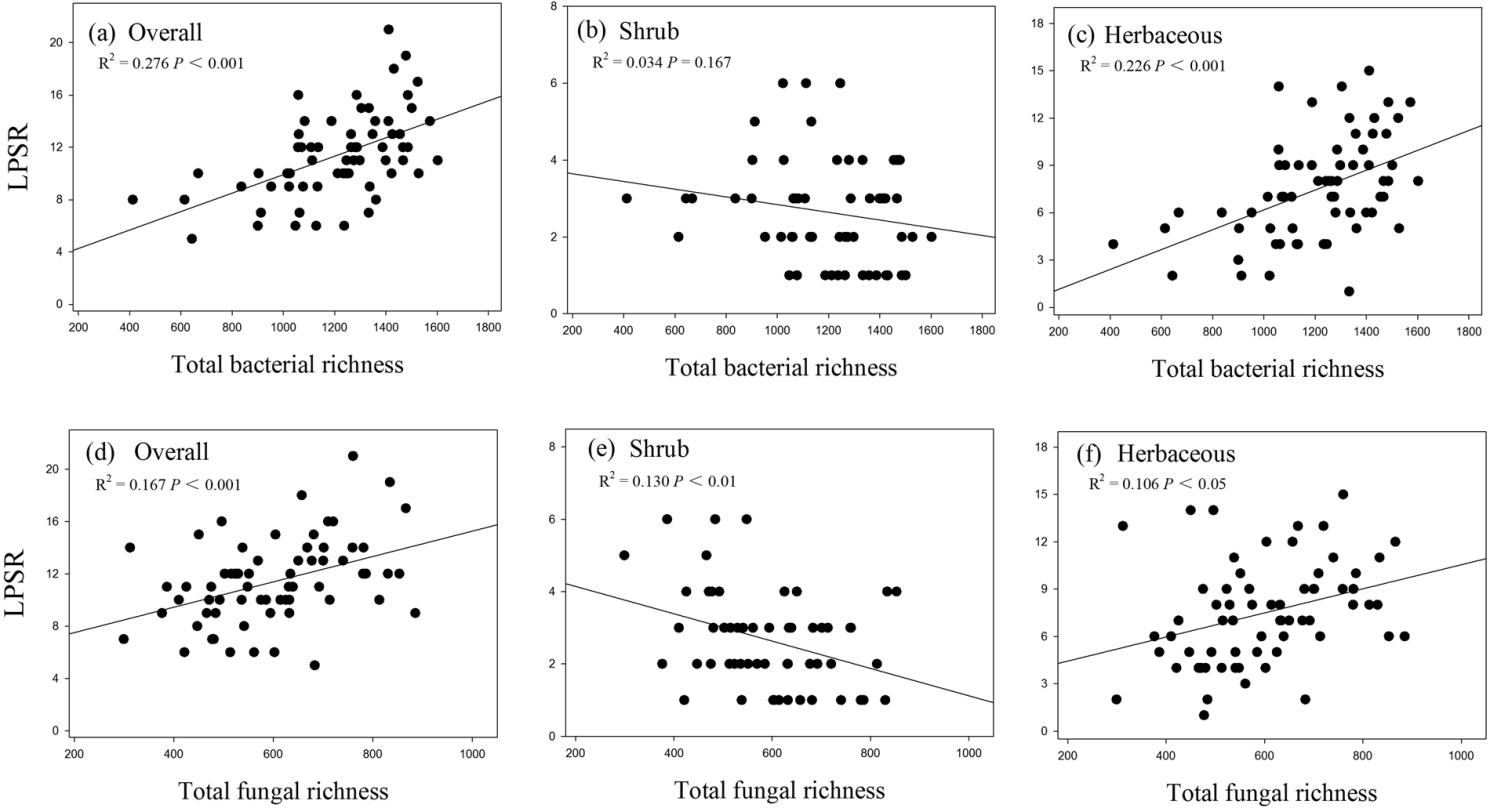
Relationships between the overall, shrub, and herbaceous LPSR and soil total bacterial (**a**–**c**) and fungal (**d**–**f**) richness.

**Figure 4:**
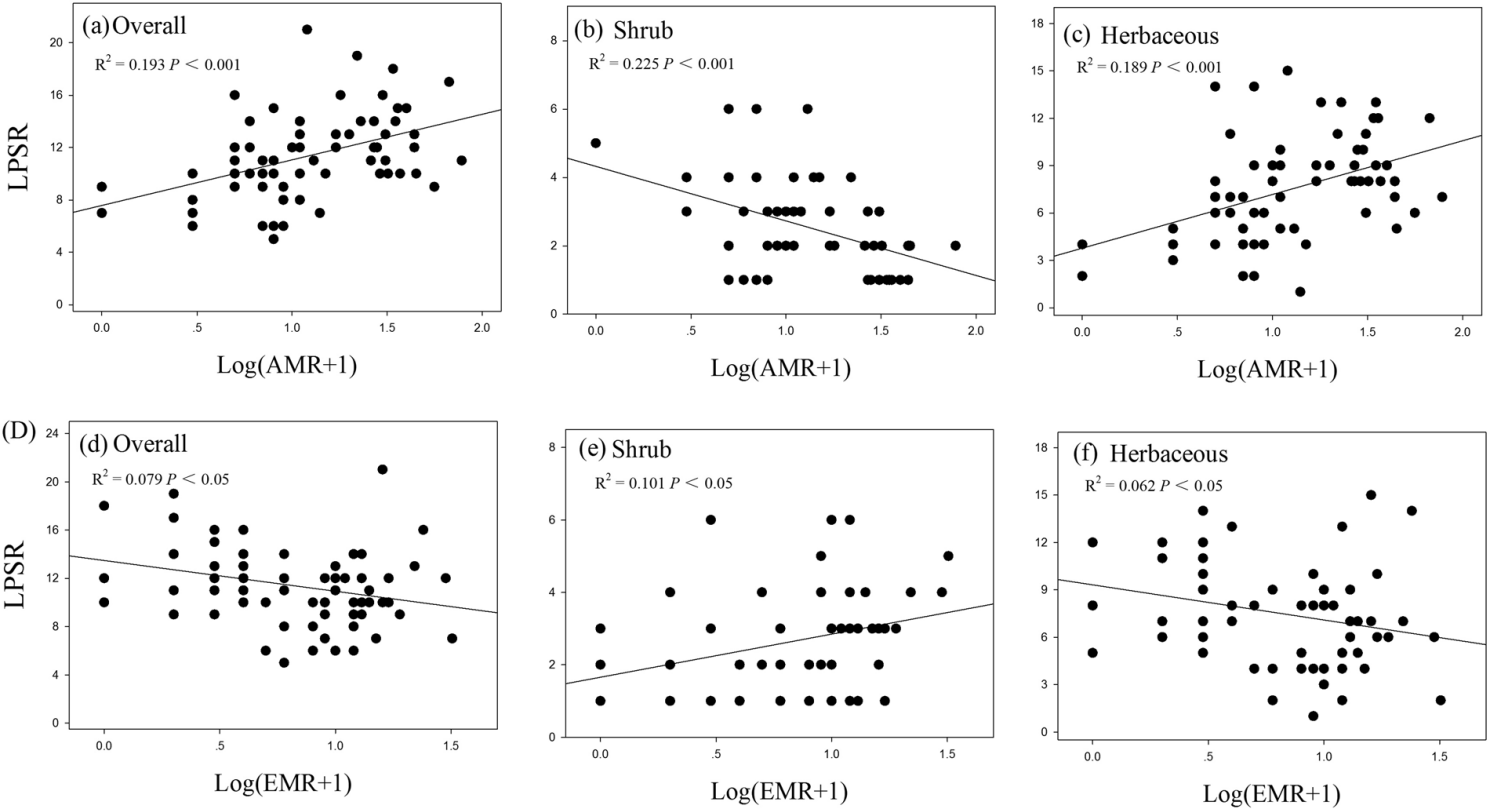
Relationships between overall, shrub, and herbaceous LPSR and soil arbuscular mycorrhizal (**a**–**c**) and ectomycorrhizal richness (**d**–**f**). Log(AMR + 1), Log (arbuscular mycorrhizal richness + 1); Log(EMR + 1), Log(ectomycorrhizal richness + 1).

**Figure 5:**
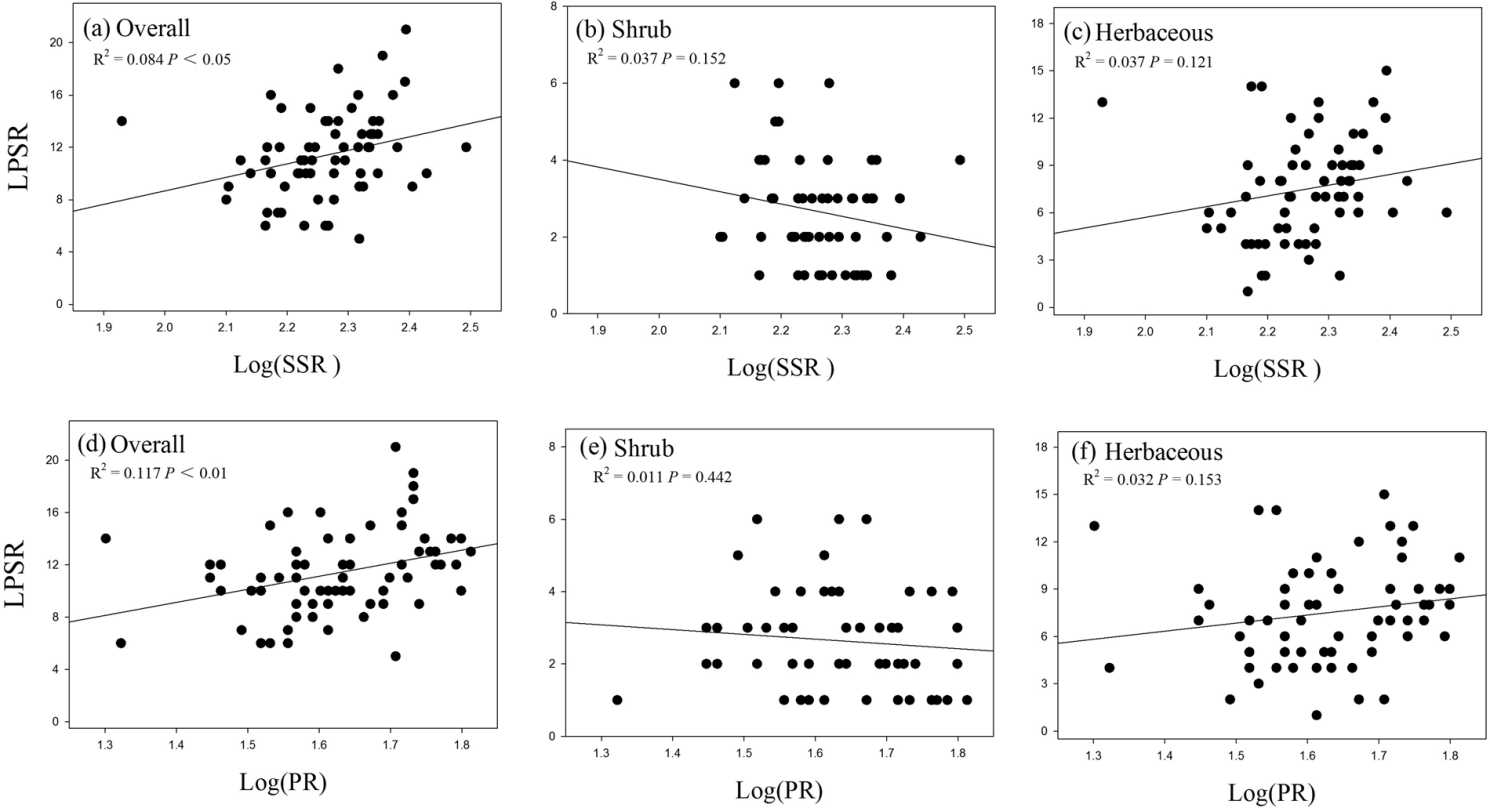
Relationships between overall, shrub, and herbaceous LPSR and soil saprotrophic (**a**–**c**) and pathogenic richness (**d**–**f**). Log(SSR), Log(saprotrophic richness); Log(PR), Log(pathogenic richness).

Both total bacterial and fungal richness, SSR, AMR and PR was positively related the overall LPSR (all *P* < 0.05; Table 3 and Figs 3, 4, and 5). The LPSR of shrub species was negatively correlated with the total fungal richness (Fig. 3(b)) and AMR (Fig. 4(a)), whereas it was positively correlated with EMR (Fig. 4(b)). The herbaceous LPSR positively correlated with total bacterial richness (Fig. 3(a)), total fungal richness (Fig. 3(b)) and AMR (Fig. 4(a)), while it negatively correlated with increasing EMR. Furthermore, the APR was uncorrelated with the LPSR for any of the groups (*P* > 0.05; Table 3), while SSR and PR were uncorrelated with the LPSR of shrub and herbaceous species (all *P* > 0.05; Table 3 and Fig. 5).

### The relative influences of climate and soil attributes on LPSR

All climatic and soil variables were used in the stepwise multivariate regression analysis (Table 4). Both climatic and soil variables were retained in the final models for overall and herbaceous LPSR, whereas all climate variables were excluded from the model for shrub LPSR. Furthermore, all climatic energy variables were excluded from the three final models.

**Table 4:** Variables retained in the regression models for explaining LPSR for overall, shrub and herbaceous species.

Species group	Variable retained in the model	R^2^	P
Overall (n = 66)	Log(SOM), TSP, MASP	0.532	<0.0001
Shrub (n = 57)	Log(TSN), Sqr(CC), Log(SOM)	0.315	<0.0001
Herbaceous (n = 66)	Sqr(CC), Log(SOM), MASP	0.592	<0.0001

TSN, total nitrogen; TSP, total phosphorus; SOM, soil organic matter; CC, clay content; MASP, mean annual summer precipitation.

**Figure 6:**
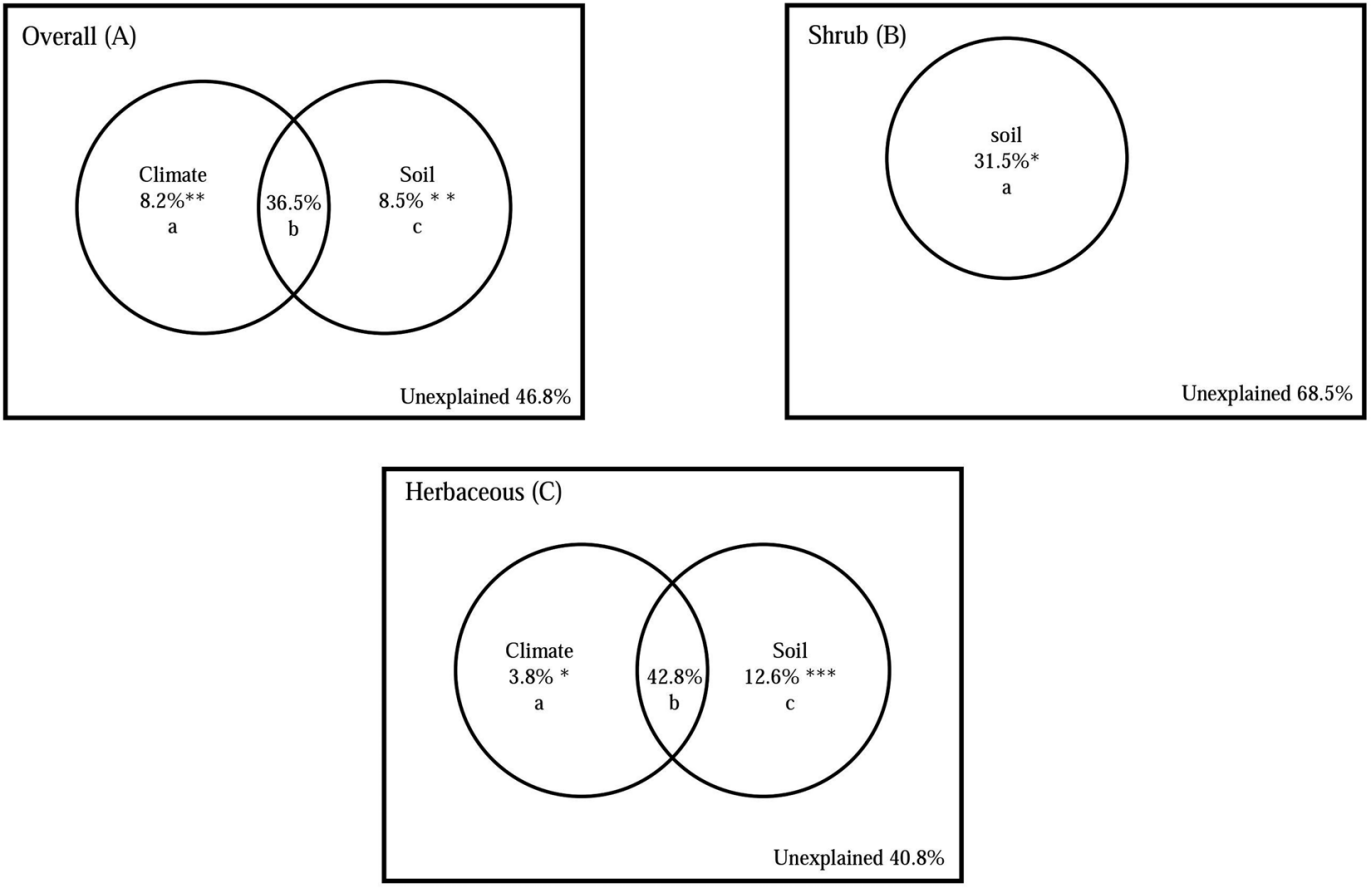
Variation partitioning for the effects of climate and soil attributes on overall (**A**), shrub (**B**), and herbaceous LPSR (**C**). The variation in LPSR in our study was decomposed into the following three fractions: pure effect of climate (a); joint effect of climate and soil attributes (b); pure effect of attributes (c); **P < 0.01; *P < 0.05; NS, *P* > 0.

A variation partitioning analysis further demonstrated that the overall and herbaceous LPSR were highly explained by climatic and edaphic variables (Table 4, Fig. 6), while the shrub LPSR was only explained by soil variables. Climate and soil attributes together explained 53.2% and 59.2% of the variation in the overall and herbaceous LPSR, respectively. However, only 31.5% of the variation in the shrub LPSR was explained by edaphic variables. When the variation was decomposed further, the pure influence of climate and soil attributes was significant for overall and herbaceous plant LPSR. However, the joint effect of climatic and edaphic factors (b) account for 68.6% and 72.3% of the total model explanatory power for overall and herbaceous LPSR, respectively

## Discussion

It has been reported that climate often strongly affects species richness^4–8,48^, but the majority of previous papers conducted at broad scales have mainly focused on species richness within geographic grids^9,10^. In particular, studies on species richness at the plot scale in the dryland of Northwest China are extremely scarce. In our study, we used data at the local scale to explore geographic patterns in LPSR in relation to climate. We found that a large proportion of the variation in the overall and herbaceous LPSR was explained by climate (44.7–46.65%; Fig. 6). Of the climatic variables, only MASP was retained in the final multiple regression model, and all climatic energy variables were excluded from the final models, although the energy variables were significantly correlated with overall and herbaceous LPSR. In contrast to studies conducted in temperate forests^11,14^, water availability is a limiting factor for plant diversity in the dryland of Northwest China. These findings support the hypothesis that in regions with a water deficit, water availability has a more important influence on plant diversity than energy availability^10,49^. However, in the dryland of China, water variables (especially MASP) were highly correlated with energy variables. For example, the correlation coefficients between MASP and MAT, MTWM, and PET were 0.532, 0.707 and 0.661, respectively (all *P* < 0.001, Figure S1), implying that the amount of water available to plants was not simply dependent on precipitation but was also strongly affected by energy input. Only AET had weak influence on shrub LPSR and all climatic variables were excluded from the final model for shrub LPSR, indicating that that climate is insufficient to explain the patterns in LPSR for shrub.

As a major carrier of exchanges of substances and energy in ecosystems, soil is the main source of the nutrients and moisture needed for plant growth and reproduction and is considered an important driving factor of plant spatial distributions^15,18,20^. Because the relationships between soil and plant diversity vary among different regions and ecosystems^21,50^, different responses of plant diversity to soil must be considered. In this study, the overall and herbaceous LPSR linearly increased with soil nutrients and moisture (e.g., SOM TSN, AN; Table 3) and linearly decreased with pH and SC. These results are consistent with those of other studies of plant diversity in arid and semiarid regions^22^.

The influences of climate and soil attributes on plant diversity have received increasing attention in recent years^14,17,21^, but few of these studies have closely quantified the relative contribution of the climate and soil to species richness patterns. Although the influence of soil attributes on plant species diversity may interact with climate^14,17^, including water and energy availability, we do not fully understand how climate and soil independently and jointly affect plant species patterns. In our study, both climatic and edaphic variables (e.g., SOM, CC; Table 4) were retained in the final multiple regression model, suggesting that soil factors are essential for explaining LPSR patterns. Climatic and soil attributes together explained 53.2%, 59.2% of the variation in overall and herbaceous LPSR, respectively (Fig. 6), while both climatic and soil factors showed significant independent effects on the overall and herbaceous LPSR. These results indicate that climatic and soil attributes together play a dominant role in determining the patterns of LPSR across the dryland ecosystem of Northwest China.

Species richness patterns differ for different functional groups^11,36,37^. In this paper, the response of the shrub LPSR to climatic and edaphic variables differed from the responses of overall and herbaceous LPSR. Climate had stronger influence on overall and herbaceous LPSR, whereas only had weak effect on shrub LPSR. Only soil variables were retained final model of shrub LPSR and explained 31.5% of the variation in shrub LPSR. The species pool hypothesis indicates that species coexist via the effects of screening by environmental factors and interspecific interactions on specific traits^51^ (e.g., drought tolerance). Because of their massive root systems, shrub species have greater drought tolerance than herbaceous species^52^. Based on our results, the patterns of overall and herbaceous LPSR are mainly shaped by climate and non-climatic factors together (e.g., soil nutrient availability), while the LPSR of shrub species is only limited by non-climatic factors. These results suggested that the LPSR of shrub species is more sensitive to local environmental factors than to climatic variables. These findings provide robust evidence that the relative roles of climate and soil attributes in controlling the patterns of LPSR differ markedly depending on the plant functional group. They further indicate that the geographic patterns plant diversity is driven by multiple factors in the dryland of Northwest China.

Both the strong, weak and no-related linkages between soil microbial diversity and plant diversity have been reported in previous studies^44,45,53–55^. In our study, total bacterial, total fungal, saprotrophic and arbuscular mycorrhizal (AM) richness were positively related to the overall and herbaceous LPSR, while animal parasites diversity negatively related to the overall and herbaceous LPSR. Moreover, total fungal and arbuscular mycorrhizal (AM) richness were negatively related to shrub richness, however, the ectomycorrhizal richness was positively related to shrub plant diversity. These results indicate that the linkages between soil microbial diversity and plant diversity may vary across functional groups of microbes and plant.

It has been shown that microbial and plant diversity can covary along significant shared gradients^56^. Plant can significantly affect microbes by host-specificity or generating diverse organic substrates^56,57^. Conversely, soil microbes may influence plant diversity through improving the nutrient availability and/or mediating plant coexistence. Microbes interact with plants through the formation of symbioses, such as with AM or EM fungi^25,52–54^. Indeed, mycorrhizal diversity or pathogen diversity can directly influence plant diversity^53,54,58^. In our study, we found that microbial diversity, especially arbuscular mycorrhizal (AM) and ectomycorrhizal richness, was significantly correlated with LPSR. These findings may imply that microbial richness significantly influences plant species richness. However, the complexity of interactions between plant and microbial diversity makes it difficult to clearly evaluate the directional effect of microbial diversity on plant diversity. Hence, the actual influence of microbes on plant diversity need deeper analyses, considering the two-way processes involved for microbes and plant diversity.

## Electronic supplementary material

Supplementary Information

Table-S2
