# Supplementary material for: Disentangling the influence of climate, soil and belowground microbes on local species richness in a dryland ecosystem of Northwest China
Source: Sci Rep. 2017 Dec 21;7:18029. doi: 10.1038/s41598-017-17860-7 (PMC5740161; doi:10.1038/s41598-017-17860-7)
Supplement: Supplementary file 1 — Supplementary Information [file 41598_2017_17860_MOESM1_ESM.pdf]

Disentangling the influences of climate, soil and belowground microbes on local species richness in a dryland ecosystem of Northwest China

Jianming Wang [1*], Ting Long[1], Yueming Zhong [1], Jingwen li[1*], Tianhan Zhang[1] Yiming Feng[2], Qi Lu[2]

1 The College of Forestry of Beijing Forestry University, Beijing Forestry University, No. 35 Qinghua East Road, Haidian District, Beijing 100083, China.

2 Institute of Desertification Studies, CAF, Beijing, NO.10 Huaishuju Road, Haidian District, Beijing, 100091. (E-mail: Lijingwenhy@bjfu.edu.cn）

* Corresponding author

Telephone number: +8601062338100.E-mail addresses: lijingwenhy@bjfu.edu.cn (J. Li)

Supplementary Table S1 List of plant species in our study

**Species**	**Famliy**	geneus	Life form
Amaranthus albus L	Amaranthaceae	Amaranthus L	annual herb
Cynanchum acutum (Willd.) Rech. f. subsp. *sibiricum* (Willd.) Rech. f.	Asclepiadaceae	Cynanchum Linn	Perennial twining vine
Arnebia fimbriata Maxim	Boraginaceae	Arnebia Forssk	perennial herb
Arnebia guttata Bge	Boraginaceae	Arnebia Forssk	perennial herb
Heliotropium ellipticum Ledeb	Boraginaceae	Heliotropium L	perennial herb
Lappula myosotis V. Wolf	Boraginaceae	Lappula V. Wolf	biennial herb
Lappula semiglabra (Ledeb.) Gurke	Boraginaceae	Lappula V. Wolf	annual herb
Lappula spinocarpos (Forssk.) Aschers	Boraginaceae	Lappula V. Wolf	annual herb
Nonea caspica (Willd.) G. Don	Boraginaceae	Nonea Medic	annual herb
Gymnocarpos przewalskii Maxim	Caryophyllaceae	Gymnocarpos Forssk	small shrub
Acanthophyllum pungens (Bge.) Boiss	Caryophyllaceae	Acanthophyllum C. A. Mey	perennial herb
Anabasis aphylla L	Chenopodiaceae	Anabasis L	small shrub
Anabasis brevifolia C. A. Mey	Chenopodiaceae	Anabasis L	small shrub
Anabasis salsa (C. A. Mey.) Benth. ex Volkens	Chenopodiaceae	Anabasis L	small shrub
Camphorosma monspeliaca L	Chenopodiaceae	Camphorosma L	small shrub
Salsola orientalis S. G. Gmel	Chenopodiaceae	Salsola L	small shrub
Salsola passerina Bunge	Chenopodiaceae	Salsola L	small shrub
Nanophyton erinaceum (Pall.) Bunge	Chenopodiaceae	Nanophyton Less	subshrub
Sympegma regelii Bunge	Chenopodiaceae	Sympegma Bunge	shrub
Krascheninnikovia ceratoides (L.) Gueldenst.	Chenopodiaceae	Ceratoides (Tourn.) Gagnebin	shrub
Iljinia regelii (Bunge) Korov.	Chenopodiaceae	Iljinia Korov	small shrub
Kalidium foliatum (Pall.) Moq.	Chenopodiaceae	Kalidium Moq	small shrub
Salsola arbuscula Pall	Chenopodiaceae	Salsola L	small shrub
Salsola laricifolia Turcz. ex Litv	Chenopodiaceae	Salsola L	small shrub
Haloxylon ammodendron (C. A. Mey.) Bunge	Chenopodiaceae	Haloxylon Bunge	small arbor
Haloxylon persicum Bge. ex Boiss. et Buhse	Chenopodiaceae	Haloxylon Bunge	small arbor
Atriplex patens (Litv.) Iljin	Chenopodiaceae	Atriplex L	annual herb
Atriplex cana C. A. Mey	Chenopodiaceae	Atriplex L	annual herb
Atriplex centralasiatica Iljin	Chenopodiaceae	Atriplex L	annual herb
Atriplex dimorphostegia Kar. et Kir.	Chenopodiaceae	Atriplex L	annual herb
Atriplex tatarica L	Chenopodiaceae	Atriplex L	annual herb
Corispermum orientale Lam	Chenopodiaceae	Corispermum L	annual herb
Corispermum lehmannianum Bunge	Chenopodiaceae	Corispermum L	annual herb
Kochia prostrata (L.) Schrad	Chenopodiaceae	Kochia Roth	annual herb
Ceratocarpus arenarius L	Chenopodiaceae	Ceratocarpus L	annual herb
Chenopodium album L	Chenopodiaceae	Chenopodium Linn	annual herb

Chenopodium prostratum Bunge	Chenopodiaceae	Chenopodium Linn	annual herb
Chenopodium serotinum L	Chenopodiaceae	Chenopodium Linn	annual herb
Dysphania aristata (L.) Mosyakin et Clemants	Chenopodiaceae	Chenopodium Linn	annual herb
Dysphania botrys (L.) Mosyakin et Clemants	Chenopodiaceae	Chenopodium Linn	annual herb
Agriophyllum squarrosum (L.) Moq	Chenopodiaceae	Agriophyllum Bieb	annual herb
Atriplex sibirica L	Chenopodiaceae	Bassia All	annual herb
Bassia dasyphylla (Fisch. et C. A. Mey.) Kuntze	Chenopodiaceae	Bassia All	annual herb
Halogeton arachnoideus	Chenopodiaceae	Halogeton C. A. Mey	annual herb
Halogeton glomeratus (Bieb.) C. A. Mey	Chenopodiaceae	Halogeton C. A. Mey	annual herb
Salsola tragus L.	Chenopodiaceae	Salsola L	annual herb
Salsola ferganica Drob	Chenopodiaceae	Salsola L	annual herb
Salsola nitraria Pall	Chenopodiaceae	Salsola L	annual herb
Salsola pellucida Litv	Chenopodiaceae	Salsola L	annual herb
Salsola affinis C. A. Mey	Chenopodiaceae	Salsola L	annual herb
Salsola brachiata Pall	Chenopodiaceae	Salsola L	annual herb
Salsola collina Pall.	Chenopodiaceae	Salsola L	annual herb
Salsola sinkiangensis A. J. Li	Chenopodiaceae	Salsola L	annual herb
Artemisia brachyloba Franch	Compositae	Artemisia Linn	small shrub
Suaeda dendroides (C. A. Mey.) Moq	Compositae	Suaeda Forsk. ex Scop	small shrub
Suaeda microphylla (C. A. Mey.) Pall	Compositae	Suaeda Forsk. ex Scop	small shrub
Asterothamnus centraliasiaticus Novopokr	Compositae	Asterothamnus Novopokr	subshrub
Cousinia affinis Schrenk	Compositae	Cousinia Cass	perennial herb
Carduus nutans L	Compositae	Carduus L	perennial herb
Saussurea japonica (Thunb.) DC	Compositae	Saussurea DC	perennial herb
Heteropappus altaicus	Compositae	Heteropappus Less	perennial herb
Crepis flexuosa (Ledeb.) C. B. Clarke	Compositae	Crepis L	perennial herb
Artemisia desertorum Spreng. Syst. Veg	Compositae	Artemisia Linn	perennial herb
Artemisia frigida Willd	Compositae	Artemisia Linn	perennial herb
Artemisia sacrorum Ledeb	Compositae	Artemisia Linn	perennial herb
Artemisia scoparia	Compositae	Artemisia Linn	perennial herb
Cirsium setosum (Willd.) MB	Compositae	Cirsium Mill. emend. Scop	perennial herb
Cichorium intybus L	Compositae	Cichorium L	perennial herb
Seriphidium borotalense (Poljak.)	Compositae	Seriphidium (Besser ex Less.) Fourr	perennial herb
Seriphidium santolinum (Schrenk) Poljak	Compositae	Seriphidium (Besser ex Less.) Fourr	perennial herb
Seriphidium terrae-albae (Krasch.) Poljak	Compositae	Seriphidium (Besser ex Less.) Fourr	perennial herb

Sonchus arvensis L	Compositae	Sonchus L	perennial herb
Echinops przewalskii Iljin	Compositae	Echinops L	perennial herb
Jurinea mongolica Maxim	Compositae	Jurinea Cass	perennial herb
Jurinea multiflora (L.) B. Fedtsch	Compositae	Jurinea Cass	perennial herb
*Taraxacum borealisinens*e Kitam	Compositae	Taraxacum F. H. Wigg	perennial herb
Taraxacum mongolicum Hand.-Mazz	Compositae	Taraxacum F. H. Wigg	perennial herb
Mulgedium tataricum (L.) DC	Compositae	Mulgedium Cass	perennial herb
Achillea ledebouri Heimerl	Compositae	Achillea L	perennial herb
Achillea wilsoniana Heimerl ex Hand.-Mazz	Compositae	Achillea L	perennial herb
Syneilesis aconitifolia (Bge.) Maxim	Compositae	Syneilesis Maxim	perennial herb
Ajania fastigiata (C. Winkl.) Poljak	Compositae	Ajania Poljak	perennial herb
Erigeron acer L	Compositae	Erigeron L	biennial herb
Artemisia sieversiana Ehrhart ex Willd	Compositae	Artemisia Linn	biennial herb
Arctium lappa L	Compositae	Arctium L	biennial herb
Arctium tomentosum Mill	Compositae	Arctium L	biennial herb
Cancrinia discoidea (Ledeb.) Poljak	Compositae	Cancrinia Kar. et Kir	biennial herb
*Artemisia marschallian*a Spreng	Compositae	Artemisia Linn	shrub
Ajania achilloides (Turcz.) Poljak. ex Grubov	Compositae	Ajania Poljak	small shrub
Ajania fruticulosa (Ledeb.) Poljak	Compositae	Ajania Poljak	small shrub
Artemisia xerophytica Krasch	Compositae	Artemisia Linn	small shrub
Xanthium mongolicum Kitag	Compositae	Xanthium L	annual herb
Artemisia blepharolepis Bge	Compositae	Artemisia Linn	annual herb
Matricaria matricarioides (Less.) Porter ex Britton	Compositae	Matricaria L	annual herb
Senecio dubitabilis C	Compositae	Senecio L	annual herb
Senecio subdentatus Ledeb	Compositae	Senecio L	annual herb
Senecio scandens Buch.-Ham. ex D. Don	Compositae	Senecio L	annual herb
Convolvulus arvensis L	Convolvulaceae	Convolvulus Linn	perennial herb
Convolvulus tragacanthoides Turcz	Convolvulaceae	Convolvulus Linn	shrub
Orostachys fimbriatus (Turcz.) Berger	Crassulaceae	Orostachys (DC.) Fisch	biennial herb
Sisymbrium loeselii L	Cruciferae	Sisymbrium L	perennial herb
Sisymbrium polymorphum (Murray) Roth	Cruciferae	Sisymbrium L	perennial herb
Dontostemon perennis C. A. Mey.	Cruciferae	Dontostemon Andrz. ex Ledeb	perennial herb
Lepidium apetalum Willd	Cruciferae	Lepidium L	annual herb
Lepidium ferganense Korsh	Cruciferae	Lepidium L	annual herb
Lepidium perfoliatum L	Cruciferae	Lepidium L	annual herb
Neotorularia torulosa (Desf.) Hedge et J. Léonard	Cruciferae	Torularia (Coss.) O. E. Schulz	annual herb
Brassica juncea (L.) Czern. et Coss.	Cruciferae	Brassica L	annual herb
Juniperus sabina L.	Cupressaceae	Sabina Mill	shrub

	Bartling		
Carex arcatica Meinsh	Cyperaceae	Carex Linn	annual herb
Carex bohemica Schreb	Cyperaceae	Carex Linn	annual herb
Carex songorica Kar. et Kir	Cyperaceae	Carex Linn	annual herb
Ephedra przewalskii Stapf	Ephedraceae Dumortier	Ephedra Tourn ex Linn	shrub
Ephedra distachya	Ephedraceae Dumortier	Ephedra Tourn ex Linn	small shrub
Euphorbia humifusa Willd. ex Schlecht	Euphorbiaceae	Euphorbia Linn	annual herb
Gentiana scabra Bunge	Gentianaceae	Gentiana (Tourn.) L	perennial herb
Geranium linearilobum DC.	Geraniaceae	Geranium L	perennial herb
Geranium pseudosibiricum J. Mayer	Geraniaceae	Geranium L	perennial herb
Geranium sibiricum L	Geraniaceae	Geranium L	perennial herb
Erodium oxyrrhynchum M. Bieb	Geraniaceae	Erodium L Her	perennial herb
Erodium stephanianum Willd	Geraniaceae	Erodium L Her	perennial herb
Koeleria macrantha (Ledeb.) Schult.	Gramineae	Koeleria Pers	perennial herb
Elymus alashanicus (Keng) S. L. Chen	Gramineae	Roegneria C. Koch	perennial herb
Achnatherum splendens (Trin.) Nevski	Gramineae	Achnatherum Beauv	perennial herb
Enneapogon desvauxii P. Beauv.	Gramineae	Enneapogon Desv. ex Beauv	perennial herb
Bothriochloa ischaemum (L.) Keng	Gramineae	Bothriochloa Kuntze	perennial herb
Leymus secalinus (Georgi) Tzvel	Gramineae	Leymus Hochst	perennial herb
Leymus tianschanicus (Drob.) Tzvel	Gramineae	Leymus Hochst	perennial herb
Bromus inermis Leyss	Gramineae	Bromus L	perennial herb
Colpodium humilie (Bieb.) Griseb.	Gramineae	Catabrosella (Tzvel.) Tzvel	perennial herb
Festuca ovina L	Gramineae	Festuca L	perennial herb
Deyeuxia arundinacea (L.) Beauv	Gramineae	Deyeuxia Clarion	perennial herb
Helictotrichon pubescens (Huds.) Pilger	Gramineae	Helictotrichon Bess	perennial herb
Cleistogenes caespitosa Keng	Gramineae	Cleistogenes Keng	perennial herb
Cleistogenes songorica (Roshev.) Ohwi	Gramineae	Cleistogenes Keng	perennial herb
Cleistogenes squarrosa (Trin.) Keng	Gramineae	Cleistogenes Keng	perennial herb
Stipa breviflora Griseb	Gramineae	Stipa Linn	perennial herb
Stipa capillata L	Gramineae	Stipa Linn	perennial herb
Stipa caucasica Schmalh	Gramineae	Stipa Linn	perennial herb
Stipa glareosa P. Smirn	Gramineae	Stipa Linn	perennial herb
Stipa grandis P. Smirn	Gramineae	Stipa Linn	perennial herb
Stipa kirghisorum P. Smirn.	Gramineae	Stipa Linn	perennial herb
Stipa macroglossa P. Smirn	Gramineae	Stipa Linn	perennial herb
Stipa orientalis Trin	Gramineae	Stipa Linn	perennial herb
Stipa purpurea Griseb	Gramineae	Stipa Linn	perennial herb
Stipa tianschanica Roshev. var. *gobica* (Roshev.) P.	Gramineae	Stipa Linn	perennial herb

C. Kuo			
Stipa tianschanica Roshev. var. *klemenzii* (Roshev.) Norl.	Gramineae	Stipa Linn	perennial herb
Agropyron cristatum (L.) Gaertn	Gramineae	Agropyron Gaertn	annual herb
Agropyron mongolicum Keng	Gramineae	Agropyron Gaertn	annual herb
Schismus arabicus Nees	Gramineae	Schismus Beauv	annual herb
Setaria viridis (L.) Beauv	Gramineae	Setaria Beauv	annual herb
Chloris virgata Sw	Gramineae	Chloris Sw	annual herb
Eragrostis pilosa (L.) Beauv	Gramineae	Eragrostis Wolf	annual herb
Poa annua L	Gramineae	Poa L	annual herb
Iris tectorum Maxim.	Iridaceae	Iris L	perennial herb
Thymus altaicus Klok. et Shost	Labiatae	Thymus Linn	small shrub
Scutellaria przewalskii Juz	Labiatae	Scutellaria Linn	subshrub
Phlomis pratensis Kar.	Labiatae	Phlomis Linn	perennial herb
Phlomis umbrosa Turcz	Labiatae	Phlomis Linn	perennial herb
Lagochilus lanatonodus C. Y. Wu et Hsuan	Labiatae	Lagochilus Bunge	perennial herb
Dracocephalum stamineum Kar. et Kir.	Labiatae	Fedtschenkiella Kudr	perennial herb
Scutellaria sieversii Bunge	Labiatae	Scutellaria Linn	perennial herb
Scutellaria baicalensis Georgi	Labiatae	Scutellaria Linn	perennial herb
Caragana dasyphylla Pojark	Leguminosae	Caragana Fabr	Dwarf shrub
Trifolium eximium Steph. ex DC	Leguminosae	Trifolium Linn	perennial herb
Trifolium repens L	Leguminosae	Trifolium Linn	perennial herb
Astragalus membranaceus (Fisch.) Bunge	Leguminosae	Astragalus Linn	perennial herb
Astragalus steinbergianus Sumnev	Leguminosae	Astragalus Linn	perennial herb
Oxytropis soongorica (Pall.) DC	Leguminosae	Oxytropis DC	perennial herb
Medicago lupulina L	Leguminosae	Medicago Linn	perennial herb
Melilotus albus Medic. ex Desr	Leguminosae	Melilotus Miller	biennial herb
Melilotus officinalis (L.) Pall	Leguminosae	Melilotus Miller	biennial herb
Caragana acanthophylla Kom	Leguminosae	Caragana Fabr	shrub
Caragana bongardiana (Fisch. et Mey.) Pojark	Leguminosae	Caragana Fabr	shrub
Caragana hololeuca Bge. ex Kom	Leguminosae	Caragana Fabr	shrub
Caragana jubata (Pall.) Poir	Leguminosae	Caragana Fabr	shrub
Caragana leucophloea Pojark	Leguminosae	Caragana Fabr	shrub
Astragalus adsurgens Pall	Leguminosae	Astragalus Linn	annual herb
Vicia sepium L	Leguminosae	Vicia Linn	annual herb
Eremurus inderiensis (M. Bieb.) Regel	Liliaceae	Eremurus M. Bieb	perennial herb
Allium flavidum Ledeb	Liliaceae	Allium L	annual herb
Allium mongolicum Regel	Liliaceae	Allium L	annual herb
Allium tenuissimum L	Liliaceae	Allium L	annual herb
Lavatera cashemiriana Cambess	Malvaceae	Lavatera Linn	perennial herb
Dicranostigma iliensis C. Y. Wu et H. Chuang	Papaveraceae	Dicranostigma Hook. f. et Thoms	perennial herb
Glaucium squamigerum Kar et Kir	Papaveraceae	Glaucium Mill	biennial herb

Hypecoum parviflorum Kar	Papaveraceae	Hypecoum L	annual herb
Plantago asiatica L	Plantaginaceae	Plantago L	annual herb
Plantago komarovii Pavl	Plantaginaceae	Plantago L	annual herb
Plantago minuta Pall	Plantaginaceae	Plantago L	annual herb
Limonium chrysocomum (Kar. et Kir.) Kuntze	Plumbaginaceae	Limonium Mill	perennial herb
Limonium sinense (Girard) Kuntze	Plumbaginaceae	Limonium Mill	perennial herb
Polygala tenuifolia Willd.	Polygalaceae	Polygala Linn	perennial herb
Rheum nanum Siev. ex Pall	Polygonaceae	Rheum L	perennial herb
Calligonum jimunaicum Z. M. Mao	Polygonaceae	Calligonum L	shrub
Calligonum mongolicum Turcz	Polygonaceae	Calligonum L	shrub
Polygonum aviculare L	Polygonaceae	Polygonum L	annual herb
Triglochin maritimum Linn	Potamogetonaceae	Triglochin Linn	perennial herb
Glaux maritima L	Primulaceae	Glaux L	perennial herb
Androsace maxima L	Primulaceae	Androsace L	annual herb
Clematis orientalis L	Ranunculaceae	Clematis L	herbaceous liane
Clematis sibirica (L.) Mill	Ranunculaceae	Clematis L	herbaceous liane
Ranunculus japonicus Thunb	Ranunculaceae	Ranunculus L	perennial herb
Thalictrum foetidum L	Ranunculaceae	Thalictrum L	perennial herb
Thalictrum minus L	Ranunculaceae	Thalictrum L	perennial herb
Aconitum apetalum (Huth) B. Fedtsch	Ranunculaceae	Aconitum L	annual herb
Aconitum nemorum Popov	Ranunculaceae	Aconitum L	annual herb
Rhamnus davurica Pall	Rhamnaceae	Rhamnus L	shrub
Potentilla angustiloba Yu et Li	Rosaceae	Potentilla L	perennial herb
Potentilla anserina L	Rosaceae	Potentilla L	perennial herb
Potentilla argentea L	Rosaceae	Potentilla L	perennial herb
Potentilla bifurca L	Rosaceae	Potentilla L	perennial herb
Potentilla reptans L	Rosaceae	Potentilla L	perennial herb
Potentilla chinensis Ser	Rosaceae	Potentilla L	perennial herb
Potentilla chrysantha Trev	Rosaceae	Potentilla L	perennial herb
Alchemilla gracilis Opiz.	Rosaceae	Alchemilla L	perennial herb
Potentilla supina L	Rosaceae	Potentilla L	biennial herb
Rosa xanthina Lindl.	Rosaceae	Rosa L	shrub
Rosa platyacantha Schrenk	Rosaceae	Rosa L	small shrub
Galium aparine Linn. var. *echinospermum* (Wallr.) Cuf	Rubiaceae	Galium Linn	perennial herb
Galium humifusum M. Bieb	Rubiaceae	Galium Linn	perennial herb
Rubia cordifolia L	Rubiaceae	Rubia Linn	perennial herb
Pedicularis songarica Schrenk	Scrophulariaceae	Pedicularis Linn	perennial herb
Pseudolysimachion incanum (L.) Holub	Scrophulariaceae	Veronica L	perennial herb
Veronica biloba L	Scrophulariaceae	Veronica L	perennial herb
Lycium ruthenicum Murr	Solanaceae	Lycium L	shrub
Tamarix chinensis Lour	Tamaricaceae	Tamarix Linn	shrub

Reaumuria kaschgarica Rupr	Tamaricaceae	Reaumuria Linn	small shrub
Reaumuria songarica (Pall.) Maxim	Tamaricaceae	Reaumuria Linn	small shrub
Carum carvi L	Umbelliferae	Carum L	perennial herb
Ferula songorica Pall. ex Schult	Umbelliferae	Ferula L	perennial herb
Urtica cannabina L	Urticaceae	Urtica L	perennial herb
Girardinia diversifolia (Link) Friis subsp. *suborbiculata* (C. J. Chen) C. J. Chen et Friis	Urticaceae	Girardinia Gaudich	annual herb
Patrinia scabiosaefolia Fisch. ex Trev	Valerianaceae	Patrinia Juss	perennial herb
Valeriana ficariifolia Boiss.	Valerianaceae	Valeriana Linn	perennial herb
Peganum harmala L	Zygophyllaceae	Peganum L	perennial herb
Zygophyllum macropodum Boriss	Zygophyllaceae	Peganum L	perennial herb
Zygophyllum fabago L	Zygophyllaceae	Zygophyllum L	perennial herb
Zygophyllum potaninii Maxim	Zygophyllaceae	Zygophyllum L	perennial herb
Zygophyllum rosovii Bunge	Zygophyllaceae	Zygophyllum L	perennial herb
*Zygophyllum rosov*ii Bunge var. *latifolium* (Schrenk) Popov	Zygophyllaceae	Zygophyllum L	perennial herb
Zygophyllum xanthoxylon (Bunge) Maxim.	Zygophyllaceae	Sarcozygium Bunge	shrub
Nitraria tangutorum	Zygophyllaceae	Nitraria L	shrub
Tribulus terrester L	Zygophyllaceae	Tribulus L	annual herb

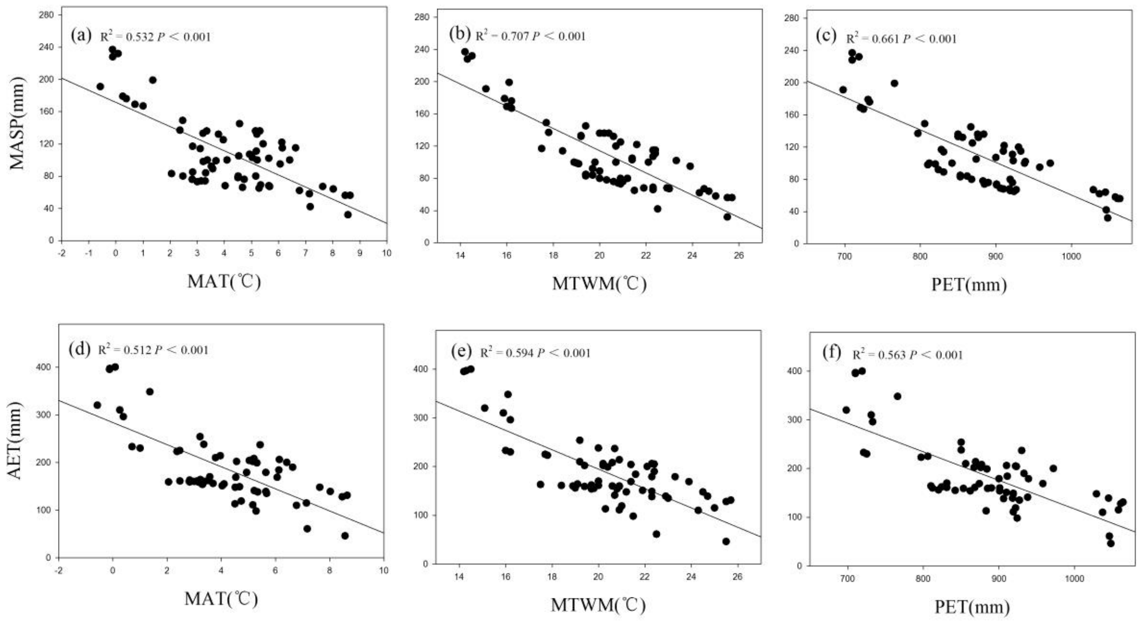

Supplementary Figure S1 Relationships between MAT, MTWM, PET and MASP (a-c), AET (d-f). MAT, mean annual temperature; PET, potential evapotranspiration; MTWM, mean temperature of the warmest month; MASP, mean annual summer precipitation; AET, actual evapotranspiration, respectively.
